# Supplementary material for: Hybrid Models and Biological Model Reduction with PyDSTool
Source: PLoS Comput Biol. 2012 Aug 9;8(8):e1002628. doi: 10.1371/journal.pcbi.1002628 (PMC3415397; doi:10.1371/journal.pcbi.1002628)
Supplement: Text S4 — Complete source code for the PyDSTool package (version 0.88.120504). Includes API documentation and help files linking to web pages. This file is identical to the current public release on Sourceforge.net. (ZIP) [file pcbi.1002628.s004.zip › PyDSTool/html/PyDSTool.Toolbox.optimizers-pysrc.html]

xml version="1.0" encoding="ascii"?


PyDSTool.Toolbox.optimizers


| Home | Trees | Indices | Help | PyDSTool |
| --- | --- | --- | --- | --- |

Package PyDSTool ::
Package Toolbox ::
Package optimizers

[hide private]

[frames] | no frames]

# Source Code for Package PyDSTool.Toolbox.optimizers

```
 1   
 2  # Matthieu Brucher 
 3  # Last Change : 2007-08-24 11:16 
 4   
 5  """ 
 6  Optimization module 
 7  """ 
 8   
 9  # Needed so that default paramaters are accessible from everywhere in the submodule 


10 -def mod_path():


11    import sys 
12    import os.path 
13    sys.path.append(os.path.abspath(os.path.dirname(__file__)))

14   
15  mod_path() 
16   
17  import defaults 
18  import criterion 
19  import line_search 
20  import optimizer 
21  import step 
22  import helpers 
23   
24  __all__= ['defaults', 'criterion', 'line_search', 'optimizer', 'step', 'helpers'] 
25   


26 -def test(level = -5, verbosity = 1):


27    from numpy.testing import NumpyTest 
28    return NumpyTest().test(level, verbosity)

29   


30 -def testall(level = -5, verbosity = 1):


31    from numpy.testing import NumpyTest 
32    return NumpyTest().testall(level, verbosity)

33
```

  


| Home | Trees | Indices | Help | PyDSTool |
| --- | --- | --- | --- | --- |

|  |  |
| --- | --- |
| Generated by Epydoc 3.0.1 on Fri May 4 15:24:19 2012 | http://epydoc.sourceforge.net |
